# Modified CAVE score for predicting late seizures after intracerebral hemorrhage

**DOI:** 10.1186/s12883-023-03510-1

**Published:** 2023-12-19

**Authors:** Yu-Ching Huang, Yi-Sin Wong, Chi-Shun Wu, Ching-Fang Tsai, Cheung-Ter Ong

**Affiliations:** 1 Department of Neurology, Tao-yuan General Hospital, Ministry of Healthy and Welfare, Tao-yuan, Taiwan; 2 Department of Industrial Engineering and Management, Yuan-Ze University, Tao-yuan, Taiwan; 3 Department of Family Medicine, Ditmanson Medical Foundation Chiayi Christian Hospital, Chiayi City, Taiwan; 4 Department of Neurology, Ditmanson Medical Foundation Chia-Yi Christian Hospital, 539 Chung-Shao Road, Chia-Yi, Taiwan; 5 Department of Medical Research, Ditmanson Medical Foundation Chiayi Christian Hospital, Chiayi City, Taiwan

**Keywords:** Stroke, Intracerebral hemorrhage, Seizure, CAVE score, Epilepsy, Anticonvulsant, Cortex

## Abstract

**Background and purpose:**

Seizures commonly occur in patients with intracerebral hemorrhage (ICH). Anticonvulsants are commonly used for preventing seizures in patients with ICH. Thus, patients with ICH at high risk of seizures must be identified. The study aims to elucidate whether double the score of cortex involvement in ICH patients can increase accuracy of CAVE score for predicting late seizures.

**Method:**

This retrospective analysis of the medical records of surviving patients admitted between June 1, 2013, and December 31, 2019. Validated the CAVE score and modified it (CAVE2). The main outcome of patients with ICH was seizures. The first seizures occurring within 7 days after a stroke were defined as early seizures. Seizures occurring after 1 week of stroke onset, including patients who had experienced early seizures or patients who had not, were defined as late seizures. CAVE and CAVE2 scores were validated using the cohort. The accuracy and discrimination of those two scores were accessed by the area under the operating characteristic curve. Akaike information criterion, integrated discrimination improvement, and continuous net reclassification improvement were used to assess the performance of the CAVE and CAVE2 scores.

**Results:**

In the cohort showed that late seizures occurred in 12.7% (52/408) of patients with ICH. Male sex, age > 65 years, cortex involvement, and early seizures were associated with the occurrence of late seizures, with odds ratios of 2.09, 2.04, 4.12, and 3.78, respectively. The risk rate of late seizures was 6.66% (17/255), 14.8% (17/115), and 47.4% (18/38) for CAVE scores ≤ 1, 2, and ≥ 3, and 4.6% (12/258), 18.3% (13/71), and 54.4 (20/37) for CAVE2 scores ≤ 1, 2, and ≥ 3 respectively. The C-statistics for the CAVE and CAVE2 scores were 0.73 and 0.74 respectively.

**Conclusion:**

The CAVE score can identify patients with ICH and high risk for late seizures. The CAVE can be modified by changing the score of cortex involvement to 2 points to improve accuracy in predicting late seizures in patients with ICH.

**Supplementary Information:**

The online version contains supplementary material available at 10.1186/s12883-023-03510-1.

## Introduction

Seizures commonly occur in patients with spontaneous intracranial hemorrhage (ICH). Seizures may occur at ICH onset within 1 week or later after a stroke. Early and late seizures were defined as appearing within 1 weeks and more than 1 weeks after ICH [1]. Studies have reported that symptomatic seizures occurred in 4–32% of patients within 2 weeks and 4–16.3% after 2 weeks of ICH onset [1–4]. The main factors that affect early seizures in patients with ICH include hemorrhage volume, cortical involvement, young age and stroke severity [4, 5]. Late seizures are long-term complications of spontaneous ICH, which may adversely affect the quality of life. The pathogenesis of late seizure is unclear; however, it may be linked to epileptogenesis following cerebral ischemia, changes in neural networks, and white and endothelial change [4]. Seizures are associated with higher mortality and poor functional outcomes in patients with ICH [6, 7]. Prophylactic antiepileptic drugs (AEDs) can lower seizure risk. Passero et al. showed that prophylactic AEDs can reduce the risk of early seizures [8–10]. Even the guidelines for the management of patients with ICH do not recommend using prophylactic AEDs for patients without seizures; [11] however, prophylactic AEDs are commonly used in patients with ICH.[12– 12] Considering the risk of seizures after ICH onset, prophylactic anticonvulsant are commonly prescribed for seizure prevention. Although anticonvulsants can reduce seizures in ICH patients, an adverse effect was also reported. Phenytoin was reported to be associated with more frequent fever and worse outcomes in patients with ICH [13]. A study reported that levetiracetam was not associated poor outcomes in patients with ICH [14]. Thus, it is important to evaluate the risks and benefits of anticonvulsants before using them in patients with ICH. Therefore, a score that can helps physicians identify patients with ICH at high risk of seizures and proper usage of anticonvulsants for seizure prevention is crucial.

The factors associated with increasing risk of late seizures include hematoma volume, cortex involvement, subdural hemorrhage, increased admission international normalized ratio, subcortical location, hematoma evacuation, and lobar brain microbleeds [2, 15–17]. To classify patients with high or low seizure risk after ICH, two scoring systems have been proposed. The CAVE score was developed 10 years ago with C-statistics of 0.81 in the study group and 0.69 in the validation group. The LANE score was developed in China 2 years ago, with C-statistics of 0.83 in the derivation group and 0.78 in the validation group [18, 19]. The CAVE score is simple and can help physicians select patients with a high risk of seizure. As ICH with cortex involvement is the most important risk factor for seizures in patients with ICH, we hypothesis that increase the score of cortex involvement in the CAVE score can improve the accuracy of the CAVE score for predicting late seizures in patients with ICH. The aim of the study was to validate the CAVE score and examine whether double the cortex involvement score (CAVE2 score) is better than the CAVE score for predicting late seizures in patients with ICH.

## Materials and methods

This is a retrospective study. Data were retrieved from the electronic medical record of a teaching hospital in central Taiwan. Between June 1, 2013, and December 31, 2019, all consecutive patients presenting with acute neurological symptoms brought to the emergency department were evaluated for stroke possibility. Patients with brain computed tomography studies showing ICH and survived at discharge were included in the study All patients with or without using anticonvulsants were included in the analysis. Patients with ICH due to head trauma, subarachnoid hemorrhage, subdural hemorrhage, ischemic stroke with hemorrhagic transformation, tissue plasminogen activator-related hemorrhage, brain tumor rupture, cerebellar and brain stem hemorrhage, and age < 20 years were excluded from the study.

All patients were evaluated by one neurosurgeon and/or neurologist. The diagnosis of seizures was based on the criteria of an international league against epilepsy [20, 21]. Motor onset focal (with or without consciousness disturbance) or generalized seizures were included in the study. Patients with nonmotor seizures were included in the study when seizures were suspected, and epileptiform discharge was confirmed by electroencephalography. Seizures occurring within 7 days after a stroke were defined as early seizures. Seizures occurring after 1 week of stroke onset, including patients who had experienced early seizures or patients who had not, were defined as late seizures [4]. Cortical involvement was considered in patients with lobar hemorrhage or patients with ICH originating from the deep brain region extending to the cortex. Hematoma volume was calculated according to the A × B × C/2 method [22].

The total CAVE score is 4 points, which included 1 point each for cortex involvement, Age < 65 years, hematoma volume > 10 ml, and early seizures. Because previous studies have shown that cortex involvement increases the risk of seizures, we hypothesized that when the cortex score was changed to 2 points, it may increase the predicted value of the CAVE score, so we changed to the CAVE2 score, which includes 2 points for cortex involvement and 1 point each for age < 65 years, hematoma volume > 10 ml, and early seizures. The receiver operating curve was used to compare the CAVE and CAVE2 scores. This study was approved by the Institutional Review Board of Ditmanson Medical Foundation Chiayi Christian Hospital Taiwan (IRB 2,020,131), and was performed in accordance with the Declaration of Helsinki (1964) and subsequent amendments. As this is a retrospective study, Ditmanson Medical Foundation Chiayi Christian Hospital IRB waived the need to obtain informed consent from each patient.

### Patient outcome

All patients with ICH hospitalization were followed up by a neurosurgeon or a neurologist. Discharge survivors were followed up at our hospital’s outpatient clinics. The primary outcome of patients with ICH was seizures. Seizures were determined by a neurologist or neurosurgeon. The study included focal-onset motor seizures with or without impaired awareness, and generalized motor seizures after reviewing all of the hospital records and outpatient records [20]. The patient was considered to have late seizures when they were diagnosed with seizures and began to use an AED after 7 days of stroke onset, had the AED dose increased, or had another AED added to the patient’s medication.

### Statistical analysis

The risk factor analysis for late seizures included sex, age, hemorrhage volume, cortex involvement, and operation which were analyzed using the Chi-square or Fisher exact test. Binary logistic regression was used to evaluate late seizure risk. The analysis was adjusted for confounding factors such as age, sex, volume, stroke history, early seizures, cortex involvement, and operation. Odds ratios were used to show the risk of each parameter.

In the next step, the receiver operation characteristics curves (ROC) and the Youden index were used to determine the optimal cutoff point for predicting late seizures by CAVE and CAVE2 scores.

We used the Akaike information criteria (AIC), integrated discrimination improvement (IDI), and continuous net reclassification improvement (NRI) to assess the performance of CAVE and CAVE2 scores.

All statistical analyses were conducted using IBM SPSS Statistics for Windows (version 28.0. IBM Corp, Armonk, NY, USA). R version 4.1.3 was used for the ROC, AIC, IDI and NRI analysis. Two-sided *P* values of < 0.05 were considered statistically significant.

## Results

Between January 1, 2013 and December 31, 2019, 701 patients were admitted because of ICH, 293 patients were excluded from the analysis, including 115 patients who died, 112 patients with brainstem and cerebellum hemorrhage, 32 patients with brain tumor, ischemic stroke with hemorrhagic transformation and subdural hemorrhage, and 34 patients loss to follow-up. A total 408 patients were included in the analysis. Table 1 shows the characteristics of the patients. Of 408 patients, 167 (40.9%) did not use anticonvulsants. Anticonvulsants were used < 1 month in 86 patients, 1–3 months in 40, 3–6 months in 10, 6 months to 1 year in 12, and > 1 year in 93. In 408 patients, 52 had late seizures, and 16 (30.8%) had seizures even though they were administered anticonvulsants.

**Table 1:** Characteristics of the patient with intracranial hemorrhage (n = 408)

Variables	No seizure (n = 356)	Late seizure (n = 52)	*p*
Sex			
Male (271)	230 (84.9%)	41 (15.1%)	
Female (137)	126 (92.0%)	11 (8.0%)	0.05
Age	63.6 ± 13.5	57.3 ± 13.5	< 0.01
< 65 (221)	185 (83.7%)	36 (16.3%)	
≥65 (187)	171 (91.4%)	16 (8.6%)	0.02
Cortex involvement			
Yes (116)	85 (73.3)%	31 (26.7%)	
No (292)	271 (92.8%)	21 (7.2%)	< 0.01
Volume (ml)	6.4 ± 13.2	19.5 ± 27.1	< 0.01
≤10 CC (240)	224 (93.3%)	16 (6.7%)	
> 10 cc (168)	132 (78.6%)	36 (21.4%)	< 0.01
Early seizure			
Yes (16)	9 (56.3%)	7 (43.7%)	
No (392)	347 (88.5%)	45 (11.5%)	0.002
Operation			
Yes (139)	104 (74.8%)	35 (25.2%)	
No (269)	252 (93.7%)	17 (6.3%)	< 0.01
Old stroke			
No (314)	272 (86.6%)	42 (13.4%)	
Ischemic stroke (57)	52 (91.2%)	5 (8.8%)	
Hemorrhagic stroke (37)	32 (86.5%)	5 (13.5%)	0.62

Under univariable analysis, age < 65 years, cortex involvement, volume > 10 ml, early seizures, and operation were associated with an increased risk of late seizure in patients with ICH. Late seizures occurred in 43.7% (7/16) of patients with early seizures, and 26.7% (31/116) of the patients had ICH with cortical involvement. Furthermore, in patients with ICH and cortical involvement and patients that had early seizures, there was a significant increase in the risk of late seizures. After adjusting for confounding factors such as sex, age, operation, cortex involvement and early seizures, binary logistic regression analysis revealed that men, age < 65 years, cortex involvement, and early seizures have a higher risk of late seizures; with odds ratios of 2.09, 2.04, 4.12, and 3.78, respectively (Table 2). In Table 3, the number of seizures in each score was listed. According to the CAVE score, the seizure rate was 6.66% (17/255) in patients who scored ≤1, the late seizure rate was 14.8% (17/115) in those who scored 2, and the late seizure rate was 47.4% (18/38) in those who scored > 2. According to the CAVE2 score, the late seizure rate was 4.6% (12/258) in patients with a CAVE2 score of ≤1, the seizure rate was 18.3% (13/71) in those with a CAVE2 score 2, and the late seizure rate was 54.1% (20/37) in those who scored > 3. A total of 22.9% (35/153) of patients with a CAVE score > 1 had late seizures, and 27.3% (27/99) of patients with a CAVE2 score > 2 had late seizures.

**Table 2:** Binary logistic regression for the risk ratio for late seizure

Parameter	Odds ratio	95% CI	*P*
Men (vs. women)	2.09	0.96–4.55	0.06
Age < 65 (vs. ≥ 65)	2.04	1.11–4.18	0.04
Operation (Yes vs. No)	4.65	2.38–9.06	< 0.001
Cortex (yes vs. No)	4.12	2.13–7.99	< 0.001
Early seizure (Yes vs. No)	3.78	1.18–12.17	0.025

CI: confidence interval

**Table 3:** Relationship between score and late seizures

CAVE Score				CAVE2 Score			
score	patient	No	LS	score	patient	No	LS
0	132	126 (95.5%)	6 (4.5%)	0	132	126 (95.5%)	6 (4.5%)
1	123	112 (91.1%)	11 (8.9%)	1	106	100 (94.3%)	6 (5.7%)
2	115	98 (85.2%)	17 (14.8%)	2	71	58 (81.7%)	13 (18.3%)
3	34	19 (55.9%)	15 (44.1%)	3	62	52 (83.9%)	10 (16.1%)
4	4	1 (25%)	3 (75%)	4	33	19 (57.6%)	14 (42.4%)
				5	4	1 (25%)	3 (75%)

LS: late seizure

**Fig. 1:**
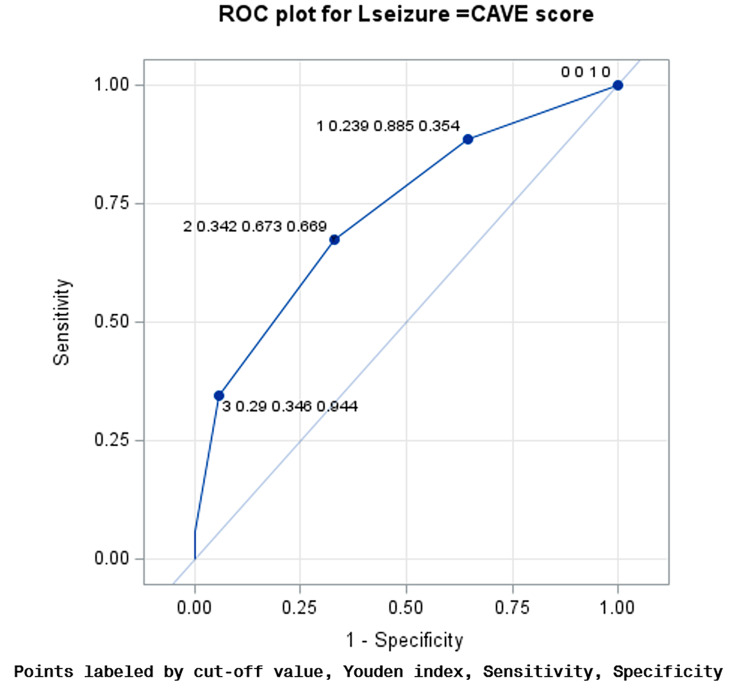
ROC curve of the CAVE score in the validation cohort. ROC, Receiver operating characteristic; AUC, area under ROC curve

**Fig. 2:**
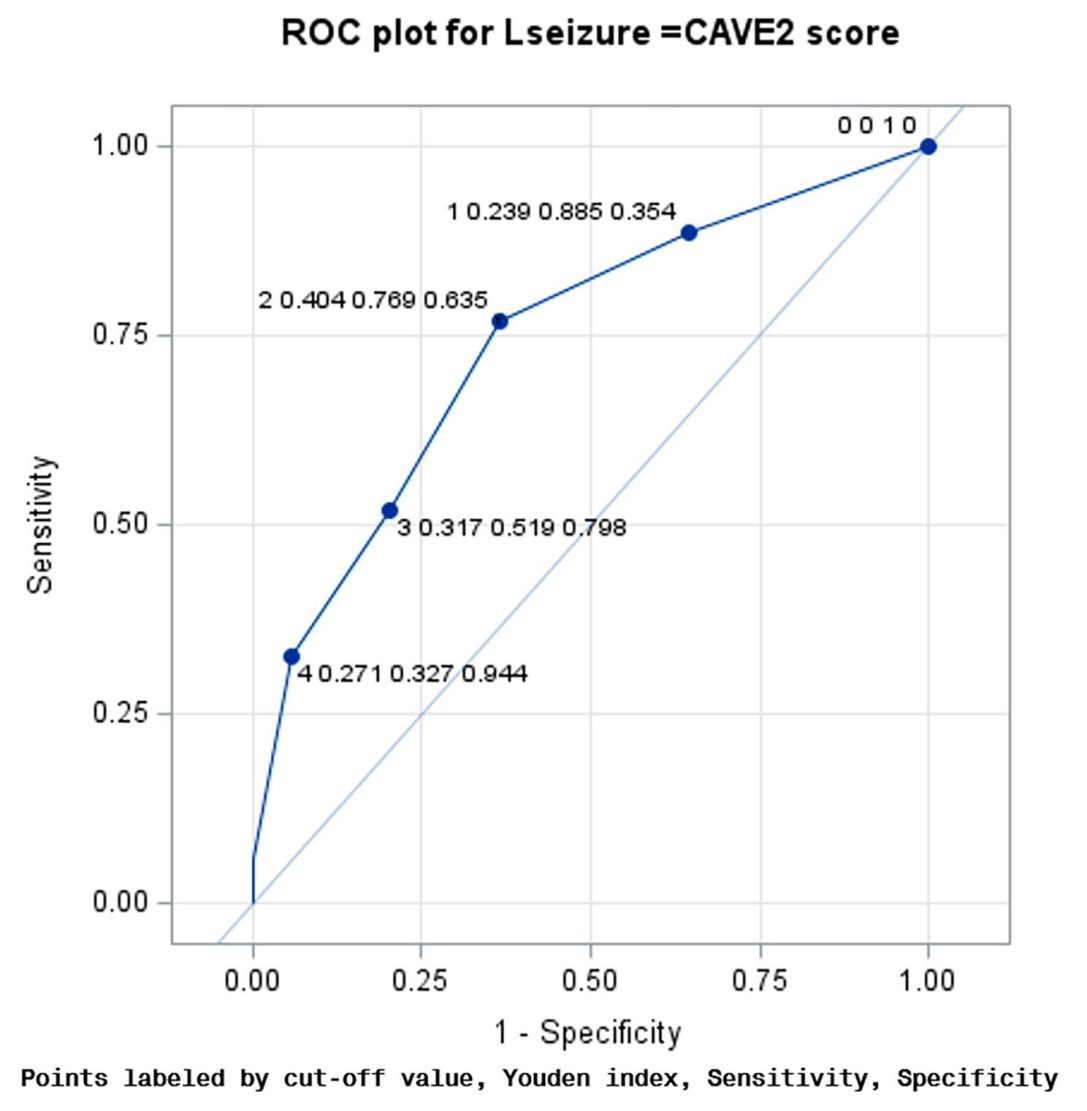
ROC curve of the CAVE2 scores in the validation cohort. ROC, Receiver operating characteristic; AUC, area under ROC curve

The C-statistics were 0.73, and 0.743 for the CAVE and CAVE2 scores respectively. For a CAVE score of 2, the ROC showed that the Youden index was 0.342, the sensitivity and specificity rates were 0.673 and 0.669 (Fig. 1), and for a CAVE2 score of 2, the Youden index was 0.404, the sensitivity and specificity rates were 0.769 and 0.635, respectively (Fig. 2). The CAVE2 score has a higher accuracy than the CAVE score for predicting late seizures. The ∆ C-statistic, IDI and continuous NRI were not significantly different between the CAVE score and CAVE2 scores (Table 4).

**Table 4:** C statistic, IDI, NRI, AIC

Score	CAVE score	CAVE2 score
C-statistic	0.730 (0.655–0.805)	0.743 (0.669–0.817)
AIC	277.307	275.782
∆C-statistic		0.013 (-0.009 ~ 0.036) (*p* = 0.250)
IDI		0.001 (-0.011 ~ 0.012) (*p* = 0.879)
Continuous NRI		0.134 (-0.145 ~ 0.412) (*p* = 0.347)

AIC: Akaike Information Criterion;

IDI: Integrated discrimination improvement;

NRI: Net reclassification improvement

## Discussion

This retrospective study analyzed patients with ICH and discovered the following: (1) late seizures are not uncommon; (2) preventive anticonvulsants in ICH patients is common; (3) anticonvulsants can reduce late seizure risk; (4) late seizure risk increases as CAVE and CAVE2 scores increase; and (5) increasing the score of cortex involvement in the CAVE score may increase seizure prediction accuracy.

Delayed seizures after intracranial hemorrhage are associated with poor outcomes. Factors associated with seizures after ICH include age, hematoma volume, cortical involvement, and genetic factors [1, 4]. Some studies have found that prophylactic anticonvulsants do not affect the outcome of patients with ICH [23]. Although the guidelines for the management of spontaneous ICH did not recommend the use of an antiepileptic agent to prevent seizures in patients with ICH [11] the use of an antiepileptic agent for seizure prevention is common in clinical practice [12, 21]. To preventing late seizures and avoid the adverse effects of anticonvulsants in patients with ICH, a physician should carefully select patients and adequately use antiepileptic agents in patients with ICH and high risk of seizures. A score that accurately identifies patients at high risk of seizures is necessary.

In our cohort the cumulative late seizure rate in our cohort at 4 years was 12.7% (52/408) in patients with ICH. The late seizure rate was in line with previous reports [18], and was higher than that reported by Wang et al. [19]. The difference was suspected to be related to our study of patients with a follow-up of 4 years and Wang et al. analyzed patients with a follow-up of 2 years. Cortical involvement was found in 28.4% (116/408) of patients in the study. This is lower than the 39% reported by Haapaniemi et al. study and higher than the 19.6% reported by Wang. In the study, 16 patients experienced late seizures during which they used anticonvulsants, which was suspected to be related to low or subtherapeutic dose of anticonvulsants [24].

During the study, we doubled the CAVE score for cortex involvement and named it the CAVE2 score. The CAVE2 score had a total of 5 points, which included 2 points for cortex involvement and 1 point each for age < 65 year, hematoma volume > 10 ml, and early seizures. Previous studies have reported that ICH with cortex involvement increases seizure risk. A 2-point score for cortex involvement may increase the predictive accuracy of the CAVE score.

Studies have shown that cortical involvement increases the risk of late seizures [2, 4, 16]. We suppose that an increase in the score for cortical involvement may increase the accuracy of the CAVE score, and we modified the CAVE score to the CAVE 2 score. The C-statistic of the CAVE and CAVE 2 scores were 0.73, and 0.743 respectively.in our validation study. The C-statistic of the CAVE2 score is higher than that of the CAVE score; however, in comparison to the CAVE score, the ∆C-statistic, IDI, and continuous NRI revealed no significant differences between the CAVE and CAVE2 scores. Some studies have found that brain surgery increases the risk of late seizures [17]. We suppose that a score including brain surgery may improve the accuracy of predicting late seizures. However, no study has investigated whether a score including craniotomy for patients with ICH may improve prediction accuracy.

Bunny et al. found the variables of the CAVE score, cortical hematoma location, age < 65 years, and hematoma volume > 10 mL plus the variables of anticoagulant use, antiplatelet use, Glasgow coma scale, international normalized ratio, and systolic blood pressure (CAV + score) can improve the accuracy for detecting early seizures [25]. Whether the CAV + is accurate in predicting late seizures is unclear. The CAVE and CAVE2 scores are superior to CAV + scores because they only include four variables and have high accuracy, which meets the criteria of a good score.

The study has the following contributions: (1) We analyzed the relationship between late seizures and CAVE2 scores. We found that over one fourth of ICH patients who have CAVE2 score over 2 will have a late seizure. (2) We also found that anticonvulsants may reduce subsequent seizures in ICH patients. (3) Furthermore, the modified CAVE core can help clinical physicians identify ICH patients with high risk of late seizures.

This study has some limitations. First, we only analyzed patients with motor seizures which may underestimate the late seizure rate. Second, only a few patients undergo electroencephalography study; however, 82.7% (43/52) of the patients received brain imaging studies to rule out the concurrent cause. Third, this retrospective study only included a relatively small number of patients from a single hospital. Fourth, our cohort did not regularly evaluate National Institutes of Health Stroke Scale, and we cannot compare the precision for predicting late seizures between CAVE, CAVE2, and LANE scores. Fifth, the study excluded many patients who lost follow-up within 1 year. However, most of those patients suffered severe strokes and were discharged to an institution. They have a high risk of mortality and seizures. To avoid underestimating late seizures, those patients were not included.

In conclusion, the CAVE2 score can identify patients with a high risk of late seizures. When patients have a high risk of late seizures and anticonvulsants are considered, we may modify CAVE by changing the score of cortex involvement to 2 points to improve accuracy in predicting late seizures in patients with ICH. Further study to investigate the effect of preventive anticonvulsants on ICH patients with high CAVE2 scores is necessary.

### Electronic supplementary material

Below is the link to the electronic supplementary material.

Supplementary Material 1

## Data Availability

All data generated or analyzed during this study are included in this published article and its supplementary information files.
