# Supplementary material for: Modified CAVE score for predicting late seizures after intracerebral hemorrhage
Source: BMC Neurol. 2023 Dec 19;23:448. doi: 10.1186/s12883-023-03510-1 (PMC10729474; doi:10.1186/s12883-023-03510-1)
Supplement: Supplementary file 1 — Supplementary Material 1 [file 12883_2023_3510_MOESM1_ESM.docx]

Supplement table: component of 3 scores

| Variable | CAVE^[21]^ | CAVE 2 | LANE^[22]^ |
| --- | --- | --- | --- |
| Cortical involvement | 1 | 2 |  |
| Age < 65 | 1 | 1 | 1 |
| ICH volume > 10 ml | 1 | 1 |  |
| Early seizure | 1 | 1 | 2 |
| Lobar bleeding |  |  | 1 |
| NIHSS ≥15 |  |  | 2 |
| Total score | 4 | 5 | 6 |

ICH: intracerebral hemorrhage
